# Improving death notification and registration: A pilot project in Lagos state, Nigeria

**DOI:** 10.7189/jogh.14.03036

**Published:** 2024-10-18

**Authors:** Samuel Keshinro, Nnamdi Orah

**Affiliations:** 1 Force Pathologist Office, Nigeria Police Force, Lagos State, Nigeria; 2 Department of Anatomic and Molecular Pathology, University of Lagos, Lagos State, Nigeria

The National Population Commission (NPopC) reports that death notification and registration are alarmingly low in Nigeria, with rates below 10% [1], despite being an important aspect of civil registration and vital statistics (CRVS). The United Nations (UN) defines CRVS as ‘the continuous, permanent, compulsory and universal recording of the occurrence and characteristics of vital events pertaining to the population in accordance with the law’ [2]. Like in many other UN member countries, birth and death registration are obligatory in Nigeria [3]. Unfortunately, unlike birth, death is mostly a sad occurrence with subsequent religious and cultural events geared towards the funeral rites without any emphasis on the obligatory notification to the NPopC, Nigeria’s statutory CRVS governmental agency.

Death notification and registration as part of CRVS are especially important because they answer questions such as:

− ‘Who died?’: this gives the legal identity of the dead, the registration of their deaths informs the government to update their records, and enables family members to inherit wealth or land.
− ‘How many died?’: this affects the census or enumeration process, and also informs death rates and potential new and emerging epidemics.
− ‘What did they die of?’: the cause of death is important for public health policy and planning.
− ‘Where did the death(s) occur?’: probable link of the death(s) to the environment or location.

Public health, population planning, and development policies will suffer without accurate data capturing of these events. CRVS and mortality data are central to monitoring several of the Sustainable Development Goals adopted by the UN, which target 80% death registration by 2030 for member nations to address under-registration [4,5].

## INTERVENTION ACTIVITIES

To strengthen death notification and registration, the Bloomberg Philanthropies Data for Health Initiative’s Global Grants Program sponsored an intervention programme implemented by the Force Pathologist Office of the Nigeria Police Force. The intervention was geared towards enhancing mortality statistics at a sub-national level in Nigeria, focussing on improving death notification and registration in Lagos State, the commercial nerve centre and the most metropolitan state of the country.

**Figure 1:**
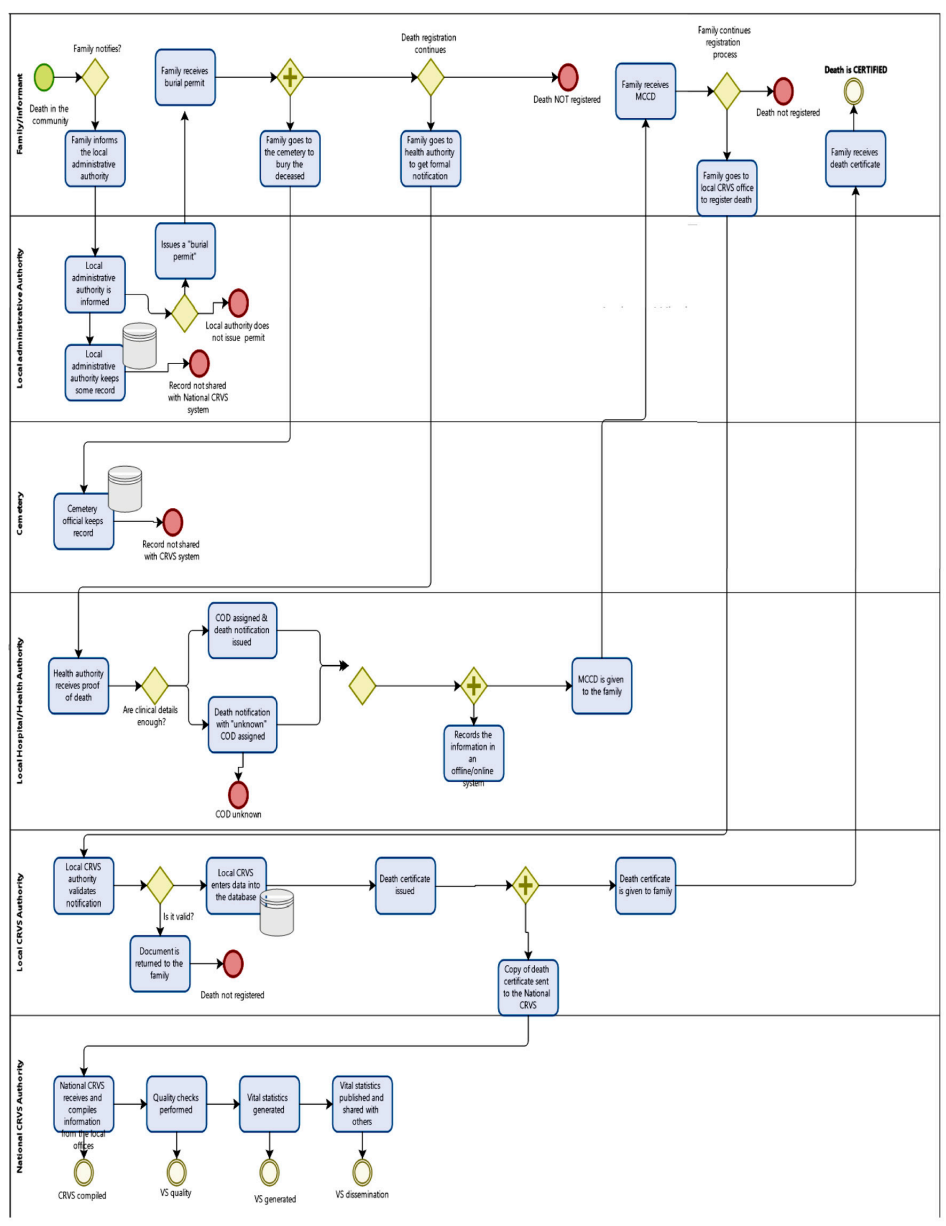
Business process mapping for notification and registration of death in the community modified from Bizagi Modeler.
− The organisation of a symposium that served as a public awareness programme and networking forum for the CRVS stakeholders, comprising the NPopC; the Lagos State Ministry of Health; the police; legislative institutions; doctor’s associations; health facilities; safety and disaster response agencies, the armed forces; the national bureau of statistics; mortuary and cemetery managers; funeral directors; traditional and religious leaders; and the media. This symposium also served as an inaugural meeting to find solutions to the bureaucracy embedded in intra- and inter-agency relationships.
− Improvement in efficiency by training personnel of NPopC, health facility records officers, and select disaster response agencies on the web-based death notification portal.
− Holding quarterly meetings for the duration of the project (one year), following up on the progress made, resolving challenges, and proposing relevant solutions.

This intervention was the first to address death notification and registration in Nigeria. It involved programmatic activities that deployed business process mapping (BPM) to identify challenges in the existing process and infused simple, sustainable information technology tools (e.g. creation of a death notification portal), public awareness, and networking among CRVS stakeholders to improve mortality data. The detailed steps of the intervention activities include:

− The development of an online, web-based death notification portal to reduce the burden associated with paper-based notification and to improve efficiency.
− The identification of the responsibilities of the individuals and government agencies involved in the death notification and registration process and the description of the different pathways (**Figure 1**).

### Intervention outcome

The 12-month-long project captured data during 2022 while using the previous year 2021 as the baseline. The combination of activities increased death notifications from 3092 to 8457 (173% increase) compared to the previous year. Furthermore, the project established a structure for continued collaboration among CRVS stakeholders by establishing institutional death notification processes, pathways for better use of data by stakeholders, and opportunities to train doctors (i.e. the obligatory death certifiers in Nigeria) and medical students (i.e. future death certifiers) on medical certifications of causes of death.

## OPINION

BPM is a process documentation technique used to visually represent a workflow to understand, interpret, and improve the existing process [6]. In this project, BPM provided insight into the engagement of the siloed stakeholders, bringing them together to develop and agree on a common understanding of the current CRVS system process. At the various meetings, significant gaps were identified in the death notification and registration process, such as the absence of institutionalised death notification, low notification of deaths that occur outside the health facilities, and the non-existence of data validation and sharing among key government agencies. These challenges were similar to those faced by other low and middle-income countries (LMICs) (e.g. Myanmar, Papua New Guinea, and Rwanda) [7].

The proposed solutions for an efficient CRVS system include centralised data aggregation, elimination of paper-based registration, and simplifying death notification and registration from health facilities. The web-based portal enabled government agencies that previously operated in isolation and lacked a formal death notification process to transmit their data collectively to the NPopC. This facilitated central storage of the data before validation. Web-based platforms for the registration of CRVS data have been successfully trialed in other countries, such as Iran and Pakistan [8]. The web-based platform also provided timely information to the state epidemiologists on public health outbreaks.

The greatest contributions to the death notification platform (84% of the total input) came from government hospitals in the state, which showed the critical role government health facilities played in the collection and transmission of data. In Bangladesh, health assistants increased death notification coverage from 4.5% to 83% [9]. Deaths that occur in the community account for about two-thirds of global deaths but constitute a challenge regarding their notification and civil registration. The low notification rates create difficulties in capturing information regarding facts and causes of death, which significantly impacts mortality statistics [7]. The project described here did not include an active system of surveillance for community deaths, which is similar to the findings in other LMICs where a passive notification process is relied upon [10]. Addressing these issues will require an extended and larger-scale intervention programme.

## CONCLUSION

CRVS systems, especially the death notification aspect in LMICs, are struggling to produce the high-quality vital statistics needed for public health policy and for monitoring and reporting on a variety of indicators. BPM can overcome the siloed treatment of these systems across the different government agencies, allowing an end-to-end view of the system as a whole in its current operations. The health sector and community leaders are vital to ensuring comprehensive and accurate collation of death notifications and data on the causes of death. In Nigeria, interventions aimed at improving knowledge about death notification have proven effective, highlighting the need to expand such initiatives. Simplifying the CRVS process, ensuring legislative backing, and providing incentives to the public will help improve death notification and the completeness of registration procedures. It is recommended that CRVS interventions of this kind will need to be institutionalised to achieve sustainable system reform.
